# cyclic AMP Regulation and Its Command in the Pacemaker Channel HCN4

**DOI:** 10.3389/fphys.2020.00771

**Published:** 2020-07-07

**Authors:** Alessandro Porro, Gerhard Thiel, Anna Moroni, Andrea Saponaro

**Affiliations:** ^1^Department of Biosciences, University of Milan, Milan, Italy; ^2^Department of Biology, Technische Universität Darmstadt, Darmstadt, Germany

**Keywords:** cyclic AMP, hyperpolarization-activated cyclic nucleotide-gated (HCN) channels, regulation, structure, “funny” current, heart rate

## Abstract

Direct regulation of the pacemaker “funny” current (I_f_) by cyclic AMP (cAMP) underlies heart rate modulation by the autonomic nervous system. At the molecular level, cAMP activates hyperpolarization-activated cyclic nucleotide-gated (HCN) channels that drive I_f_ in sinoatrial node (SAN) myocytes. Even though HCN channel genes were identified more than 20 years ago, the understanding of how cAMP regulates their gating is still fragmented. Here we summarize present understanding on how the cAMP signal is transmitted from the cytosolic to the transmembrane (TM) domain in HCN4. We further discuss how detailed structural knowledge prompted the development of pharmacological/genetic tools for the control of cAMP regulation in these channels.

## Introduction

Automaticity of heartbeat originates in the sinoatrial node (SAN), where specialized cardiomyocytes initiate spontaneous impulses in the absence of external stimuli. A main player of cardiac automaticity is the “funny” current (I_f_) that, unique among voltage-gated channels, is activated by hyperpolarization of membrane voltage. I_f_ is a mixed Na^+^/K^+^ inward current that slowly depolarizes pacemaker cells to the threshold for action potential firing (DiFrancesco, 1993). In addition to voltage, I_f_ is modulated by the second messenger cyclic AMP (cAMP), which enhances channel open probability, shifting the voltage-dependency of opening to more positive values and increasing the amount of current at any given voltage (DiFrancesco and Tortora, 1991). This mechanism is of crucial physiological relevance as it contributes to the autonomic regulation of heart rate by adrenaline and acetylcholine, which modulate cAMP concentration of SAN myocytes. It is indeed worth noting that cAMP controls different pathways in SAN myocytes, all converging to the modulation of heart rate (Behar et al., 2016).

The molecular determinants of I_f_ are hyperpolarization-activated cyclic nucleotide-gated (HCN) channels (Santoro et al., 1998; Ludwig et al., 1999; Wainger et al., 2001). HCN channels are encoded by four distinct genes in mammals (HCN1–HCN4). The four closely related isoforms each express a hyperpolarization-activated cation current, whose kinetics range from fast (HCN1) to very slow (HCN4). HCN isoforms also differ in their voltage-dependence with HCN1 opening at the most depolarized and HCN4 at the most hyperpolarized potentials (as assessed by their half-activation voltage or V_1/2_ value). The channels further differ in their cAMP response with HCN1 displaying the smallest and HCN4 the largest maximal voltage shift with saturating concentrations of ligand (Wahl-Schott and Biel, 2009). HCN4 is the main subunit expressed in SAN (Brioschi et al., 2009), and the characteristic properties of HCN4, slow activation kinetics and the strong response to cAMP, closely match those of I_f_ (Baruscotti and Difrancesco, 2004). Noteworthy, HCN1 and HCN2 have been found in the SAN as well, but the levels of expression are usually much lower compared to HCN4 and vary a lot among different species. Despite their poor presence, there is evidence that they contribute to the cardiac pacemaking I_f_ (Herrmann et al., 2011; Bucchi et al., 2012; Fenske et al., 2013).

As for the role of HCN in heart rate acceleration during sympathetic stimulation, there are controversial reports on the role of HCN4. Two initial studies performed in mice deleted of HCN4 in a temporally controlled manner showed that the cAMP regulation of HCN4 is not required for the modulation of the heart rate, as the ECG recordings in freely moving HCN4 knockout (KO) mice did not reveal major alterations of the cardiac electrical activity (Herrmann et al., 2007; Hoesl et al., 2008). However, further studies highlighted the essential role of HCN4 in establishing the basal heart rate and its cAMP-dependent regulation and in reaching the maximal heart rate (Alig et al., 2009; Baruscotti et al., 2011). Therefore, the relevance of HCN4 for the regulation of heart rate is clear, though HCN4 may not contribute to all the phenomena involved in this process. The role of HCN4 in the different levels of modulation of heart rate has been already analyzed in detail in a review (Bucchi et al., 2012) and it is beyond the purpose of our review, which, as already stated, is specifically focused on the molecular details of cAMP regulation of HCN4.

Here, we review current knowledge on how cAMP binds to HCN channels and affects their gating mechanism. Taking HCN4 as a paradigm, we will further show how the precise knowledge of this mechanism leads to the discovery of modalities to interfere with it, paving the way to future therapeutic and pharmacological interventions for the control of heart rate.

## Insight into cAMP Regulation Comes from the Structures of HCN Channels

To date, the available structural information on HCN channels include the full length model of HCN1 (Lee and MacKinnon, 2017, 2019) and several detailed structures of the cytosolic domain, which host the cAMP binding sites of HCN4, HCN2, and HCN1 (Zagotta et al., 2003; Xu et al., 2010; Lolicato et al., 2011, 2014; Akimoto et al., 2014; Saponaro et al., 2014).

The full length structure of the human HCN1 channels was recently solved using cryo-electron microscopy single particle analysis (cryo-EM) in the presence and absence of bound cAMP (Lee and MacKinnon, 2017, 2019). The overall structure of HCN channels is similar to that of other members [EAG (Whicher and MacKinnon, 2016) and hERG (Wang and MacKinnon, 2017)] of the superfamily of voltage-gated K^+^ channel to which HCN belongs.

**Figure 1:**
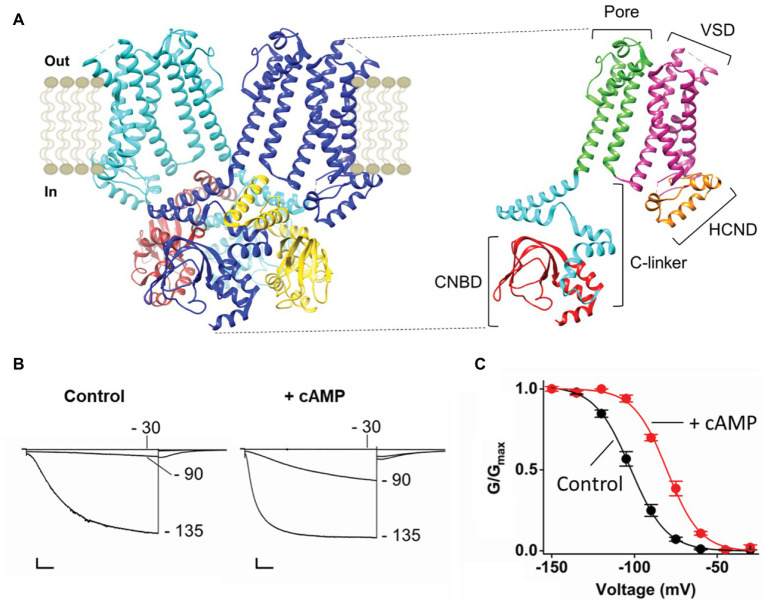
**(A)** Left, ribbon representation (side view) of two opposite subunits of HCN1 (PDB_ID: 5U6O) colored in blue and light blue. The four cytoplasmic C-terminal regions of HCN1 are shown and colored in blue, brown, light blue, and yellow. Right, ribbon representation (side view) of one subunit of HCN1. The domains of the protein are labeled: The Cytoplasmic N-terminal hyperpolarization-activated cyclic nucleotide-gated domain (HCND) in orange, the voltage sensor domain (VSD) in magenta, the pore domain (PD) in green, the cytoplasmic C-terminal C-linker in light blue, and the cyclic nucleotide binding domain (CNBD) in red. **(B)** Representative whole-cell currents of HCN4 recorded at the indicated voltages in control solution (left) and in the presence of saturating cyclic AMP [cAMP; right; adapted from Porro et al. (2019) with permission]. Scale bar is 200 pA × 500 ms. **(C)** Mean activation curves of HCN4 in control solution (black) and in the presence of saturating cAMP [red; adapted from Porro et al. (2019) with permission]. Values of the activation curves are shown as mean ± SEM.

HCN channels are composed of four subunits assembled around a central pore. Figure 1A displays only two opposite subunits in the membrane and four in the cytosolic side, for clarity. Each monomer is composed of six transmembrane (TM) domains, of which S1–S4 form the voltage sensor domain (VSD) and S5–S6 form the pore domain (PD) that hosts the selectivity filter (SF). The N and C termini are cytosolic, the N-terminus contains the HCN domain (HCND) and the C terminus contains the cyclic nucleotide binding domain (CNBD), which is connected to the S6 in the PD *via* a C-linker. The C-linker contains two helix-turn-helix motifs (formed by helices A', B' and C', D', respectively) that form the gating ring of the tetramer by resting as “an elbow on the shoulder” of the neighbor subunit (Zagotta et al., 2003).

Binding of cAMP occurs at the CNBD and initiates the signal that is transmitted *via* the C-linker to the TM part of the channel, where it increases pore open probability by reducing the energetic request of the VSD. Figure 1B shows the response of HCN4 currents to the addition of saturating concentrations (30 μM) of cytosolic cAMP. Ligand binding increases the amount of current recorded at intermediate voltage (−90 mV in this example) without affecting the maximal current recorded at saturating voltage (−135 mV). This effect of the ligand on voltage-dependency of the channel is reflected in a right shift (about 20 mV) of the half activating voltage (V_1/2_), as shown in the activation curve that describes channel open probability as a function of voltage (Figure 1C).

The comparison of the available HCN1 structures, obtained in the cAMP-free (apo) and -bound (holo) form, provides little mechanistic information on how pore gating may be modulated by cAMP. Minimal differences in the conformation of the C-linker are observed between the apo and holo structures of HCN1 (Lee and Mackinnon, 2017), despite a large body of literature arguing that movements of the CNBD transfer force to the TMD portion of the channel through a rearrangement in the C-linker, leading to a rotation of the elbow (Craven and Zagotta, 2004; Craven et al., 2008; Weissgraeber et al., 2017; Gross et al., 2018). This finding is somehow not surprising considering the known minimal response of HCN1 to cAMP (Porro et al., 2019), and highlights the expectation for other structures of HCN isoforms with a larger cAMP response to come.

Present knowledge on the intramolecular pathway of cAMP effect in HCN channels, thus relies on a large amount of previous work performed on the isolated cytosolic portion of the channel that comprises only the CNBD or the C-linker/CNBD (Zagotta et al., 2003; Xu et al., 2010; Lolicato et al., 2011, 2014; Puljung and Zagotta, 2013; Akimoto et al., 2014; Puljung et al., 2014; Saponaro et al., 2014). It is nonetheless to be mentioned that, despite the above numerous studies showing that the cAMP modulation in HCN is mediated by direct binding of the molecule to the CNBD, it has been reported that the channel can be also regulated by a cAMP-dependent mechanism through protein kinase A (PKA; Liao et al., 2010).

## The Conformational Changes Induced by cAMP in the CNBD

**Figure 2:**
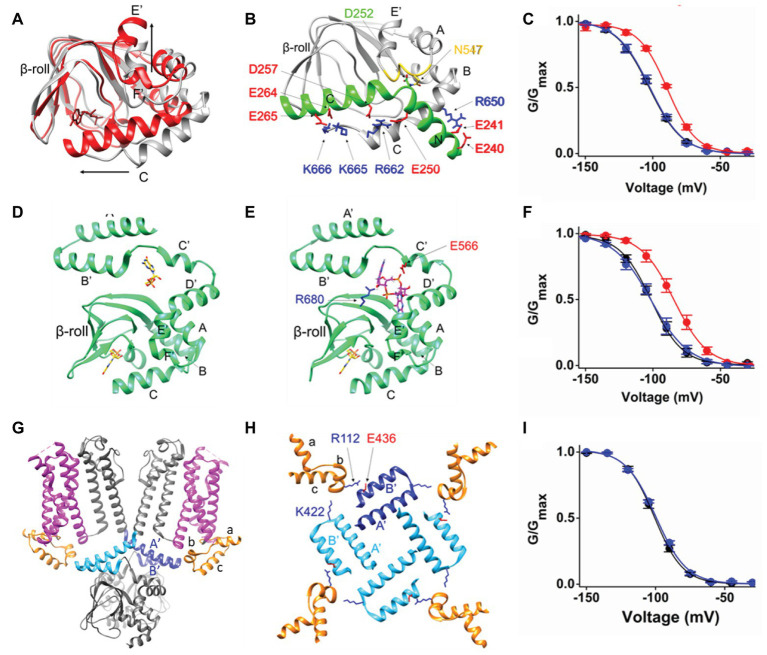
**(A)** Superimposition of the two structures of HCN2 CNBD, represented as ribbons, solved in the absence (gray, PDB_ID: 2MPF) and in the presence of cAMP (red, PDB_ID: 3U10). cAMP, represented as red sticks, is shown within the beta roll. The structural elements involved in the principal movements induced by cAMP are labeled. The black arrows indicate the direction of such movements. **(B)** Ribbon representation of HCN2 CNBD (gray) in complex with TRIP8b_nano_ peptide [green; adapted from Saponaro et al. (2018) with permission]. The structural elements of both proteins are labeled. The N-bundle loop of the CNBD is colored in yellow. The crucial residues involved in the interaction are shown as sticks and labeled. In particular, the negative residues of TRIP8b_nano_ are colored in red, while in blue the positive residues of HCN2 CNBD. **(C)** Mean activation curves of HCN4 in control solution (black), in the presence of cAMP (red) and in the presence of cAMP and TRIP8b_nano_ [blue; adapted from Saponaro et al. (2018) with permission]. **(D)** Ribbon representation of HCN4 C-linker/CNBD (green) solved in the presence of cGMP (PDB_ID: 4KL1). The two cyclic GMP (cGMP) molecules, represented as sticks, are shown and colored in yellow. **(E)** Docking simulation of c-di-GMP (magenta stick) molecule in the structure of HCN4 C-linker/CNBD (green) solved in the presence of cGMP [PDB_ID: 4KL1; adapted from Lolicato et al. (2014) with permission]. The crucial residues involved in the interaction with c-di-GMP are shown, labeled and colored based on their chemical nature: blue and red for the positively and negatively charged residues, respectively. The cGMP molecule bound to CNBD is represented as sticks and colored in yellow. **(F)** Mean activation curves of HCN4 in control solution (black), in the presence of cAMP (red) and in the presence of cAMP and c-di-GMP [blue; adapted from Lolicato et al. (2014) with permission]. **(G)** Ribbon representation (side view) of two opposite subunits of HCN1 (gray, PDB_ID: 5U6O). The HCNDs are colored in orange, the VSDs are colored in magenta, and the C-linker “elbow” elements (A' and B' helices) are colored in blue and light blue in the two subunits. **(H)** Top view of the HCNDs (orange) and the C-linker “elbow” elements (one subunit in blue and the other three in light blue). The structural elements forming the HCND and the C-linker “elbow” elements are labeled. The residues involved in the interaction between the HCND and two adjacent C-linker “elbows” are shown as sticks, labeled and colored-coded based on their chemical nature as in panels **(B,E)**. **(I)** Mean activation curves of HCN4 wt (black) and K543A-E557A double mutant [blue; adapted from Porro et al. (2019) with permission]. Values of the activation curves shown in panels **(C,F,I)** are shown as mean ± SEM.

The CNBD has the conserved motif of a per-ARNT-sim (PAS) domain composed by a beta roll (beta sheets 1–7) and alpha helices (E', F', A, B, P, and C). It contains a cAMP binding pocket at the interface between the beta roll and the alpha helices. Binding of cAMP induces most changes in the C-helix. The C-helix undergoes a translational movement toward the beta roll and establishes crucial contacts with the cAMP moiety. During this process the C-terminal end of the C-helix folds and the overall length of the helix increases, thus becoming a sort of a “lid” on the binding pocket. The other relevant movement occurring in the CNBD upon cAMP binding is the upward displacement of helices E' and F' (Saponaro et al., 2014). These two helices are directly connected to the C-linker. Their movement thus transmits the cAMP signal to the C-linker, initiating the conformational changes that will eventually affect pore opening. It is therefore expected that blocking cAMP-induced movements in the CNBD will prevent the effect of the ligand on the current. Indeed, in the nervous system, HCN channels have a cytosolic regulatory subunit, TRIP8b, that inhibits channel response to cAMP by physically interacting with the CNBD (Santoro et al., 2004; Hu et al., 2013; Saponaro et al., 2014, 2018). The minimal portion of TRIP8b that recapitulates the effect of the full length protein on channel gating is 40 residues long (named TRIP8b_nano_) and comprises two short alpha helices, which fold upon binding to the CNBD (Saponaro et al., 2018). The structural model of the complex between HCN2 CNBD and TRIP8b_nano_ was obtained by NMR, and is shown in Figure 2B. TRIP8b_nano_ (in green) binds to the CNBD in the cAMP-unbound (apo) state (in gray) and its interaction is predominately driven by electrostatic contacts with the CNBD C-helix.

The structural model of the complex fully explains the articulated mechanism through which TRIP8b antagonizes cAMP binding to the CNBD. cAMP and TRIP8b share the same binding sites on the C-helix and this accounts for the direct competition previously highlighted by functional studies (Han et al., 2011; Deberg et al., 2015; Bankston et al., 2017). On the other hand, TRIP8b interacts with the loop between E'-F' helices (N-bundle loop, colored in yellow in Figure 2B), which constitutes an allosteric regulative element for cAMP binding (Saponaro et al., 2018). This accounts for the allosteric inhibition component of the antagonistic action of TRIP8b on the cAMP effect previously proposed (Hu et al., 2013).

Thus, even though cAMP can still bind to the pocket inside the beta roll, the presence of TRIP8b_nano_ prevents cAMP-induced movements in the C-helix and in the E'-F' helices (shown in Figure 2A), thus inhibiting cAMP effect on the current (Figure 2C).

TRIP8b_nano_ has been used as a peptide tool to selectively prevent adrenergic regulation of I_f_ in SAN myocytes, reducing the action potential firing rate in isolated myocytes by about 30% (Saponaro et al., 2018). TRIP8b_nano_ is also available in a cell-penetrating form, obtained by fusing the viral TAT peptide sequence at its N-terminus (Saponaro et al., 2018). In this form, TRIP8b_nano_ can be added directly to the external solution, widening its range of applications as a research tool as well as a potential peptide drug whenever the response of HCN channels to cAMP needs to be dampened.

## Transmission of cAMP Effect Through the C-Linker

X-ray crystallography has been successfully used to obtain the structure of the cytosolic portion of HCN channels that include the C-linker and the CNBD (C-linker/CNBD; Zagotta et al., 2003; Xu et al., 2010; Lolicato et al., 2011, 2014). In these structures, the C-linker adopts a tetrameric configuration known as “elbow on shoulder,” in which the elbow of one subunit, formed by helices A' and B', rests on the shoulder, formed by helices C' and D' of the adjacent subunit. Such a configuration has been confirmed by the full length structure of HCN1 (Lee and MacKinnon, 2017, 2019). Several hydrophilic and hydrophobic contacts connect the elbow and the shoulder creating a network of interactions that can easily propagate the CNBD-initiated movements toward the TM domains. In the specific case of HCN4, in which the C-linker/CNBD was crystallized in the presence of the agonist cyclic GMP (cGMP), the ligand was found to be bound to a second pocket, besides the canonical ligand binding site in the CNBD (Lolicato et al., 2014). This result led to the discovery of a regulatory pocket at the interface between the CNBD and the C-linker, the C-linker pocket (CLP; Figure 2D). *In silico* docking performed in the CLP, identified a class of potential binders, cyclic-di-nucleotides (c-di-nucleotide; Figure 2E). This is a class of regulatory molecules originally discovered in bacteria (c-di-GMP and c-di-AMP) but more recently found also in mammals (cGAMP; Wu et al., 2013). When tested in patch clamp experiments, c-di-GMP, c-di-AMP, and cGAMP, all efficiently prevented cAMP effect in HCN4 channels with micromolar (c-diGMP and c-di-AMP) and submicromolar (cGAMP) affinity (Lolicato et al., 2014). Several mutations introduced in the CLP, prevented c-di-nucleotide action allowing normal cAMP response of the mutants channels (Figure 2F; Lolicato et al., 2014). It is believed that the occupancy of the CLP by c-di-nucleotides blocks the transmission of the movement from the CNBD to the C-linker inasmuch as the CLP corresponds to the flexible link between the two movable parts of the C-linker, the elbow and the shoulder. In this way, the upward movement of the E' and F' helices of the CNBD (see Figure 2A) cannot be converted into the expected rotation of the upper part of the C-linker.

Notably, this effect is isoform-specific as only HCN4 responds to c-di-nucleotides (Lolicato et al., 2014). This is remarkable, given the high degree of amino acid conservation in the critical residues of the CLP among different HCN subunits. At the same time, this is very exciting because c-di-nucleotides constitute the only HCN-specific inhibitors available so far. C-di-nucleotides have been shown to reduce cAMP response and firing rate in isolated myocytes of the SAN of mouse (Lolicato et al., 2014). It is worth noting that, even though there is no direct evidence of c-di-nucleotides in the heart, it is known that the key enzyme for cGAMP production, cyclic-GMP-AMP synthase (cGAS), is highly expressed in human cardiac tissues (Uhlen et al., 2010). Moreover, it was demonstrated that c-di-nucleotides can be also transferred from producing cells to neighboring cells through gap junctions, thus rapidly spreading them in a horizontal manner (Ablasser et al., 2013). So far, c-dinucleotides as second messengers in mammals have been associated to the immune system (Li et al., 2013), but the findings reported above allow to hypothesize a possible role of endogenous c-di-nucleotides in the control of I_f_, either because of their direct production in cardiomyocytes, or because of their spreading *via* gap junctions following the activation of the immune response.

## The Transmission of the cAMP Effect to the TM Domain

The full length structure of HCN1 has shown for the first time in the presence of three helical folded domains (helices A–C, see Figures 2G,H), termed HCND, at the cytosolic N-terminus of the channel, right before TM1 (Lee and MacKinnon, 2017). In the 3D structure, the HCND wedges in between the C-linker and the VSD possibly establishing contacts with both of them (Figure 2G). In a recent work, Porro et al. (2019) highlighted the network of hydrophobic and hydrophilic interactions that physically connect the HCND to the C-linker and the VSD, and underscored their crucial role in mechanically transmitting the cAMP effect from the cytosolic to the TM domain (Porro et al., 2019). Figure 2H shows a top view of the tetrameric arrangement of the HCND and the C-linker. Each HCND, in orange, establishes, *via* the top part, hydrophobic contacts with the VSD, in magenta, of its own subunit in the membrane (not shown) and with the lower part electrostatic interactions with the C-linkers of the adjacent (light blue) and opposite (blue) subunits. The HCND thus forms a physical continuum between the cytosolic C-linker and the TM VSD domain.

Mutations of the two resides on the C-linker, E436 and K422 (human HCN1 numbering), completely abolish the response of the channel to cAMP, leaving the properties of the channel unaltered. This result was reproduced in all HCN isoforms tested, HCN1, HCN2, and HCN4 (Figure 2I shows the effect of the double mutation in HCN4; Porro et al., 2019), confirming the hypothesis that the HCND is involved in the transmission of the cAMP-induced movement from the cytosolic to the TM domain. In particular, on the basis of molecular dynamic (MD) simulations and linear response theory calculations, it was concluded that the HCND acts as a transmission crank converting the circular rotation of the C-linker into a vertical displacement in the VSD (Porro et al., 2019).

These data provide structural-based support to the view that the C-linker must be physically connected to the VSD to transmit cAMP-induced movements originating in the CNBD. Experimental evidences came originally from a study of Kusch et al. (2010) showing that, in HCN2, the movement of the voltage sensor changes the affinity for cAMP in the CNBD without involving the PD. This result was obtained by simultaneous recording of currents and fluo-cAMP binding, a technique that goes under the name of patch-clamp fluorometry.

Even though the precise allosteric pathway is not yet defined in full detail, it is nonetheless expected that this communication pathway works in both directions, and that the signal of the presence of cAMP and the signal of the presence of voltage will travel along the same pathway through the HCN tetramer, although in opposite directions.

In conclusion, recent advancement in the comprehension of how cAMP regulates gating in HCN channels came from structural studies of the full length as well as from cytosolic portions of the channel. Reconstruction of the allosteric pathway from the CNBD to the VSD allows the control and the command over cAMP effect in the channel providing new tools for research and future pharmacological intervention.

## Author Contributions

All authors critically reviewed the literature. AM and AS wrote the text and prepared the figures. All authors contributed to the article and approved the submitted version.

### Conflict of Interest

The authors declare that the research was conducted in the absence of any commercial or financial relationships that could be construed as a potential conflict of interest.
